# Intra-action review of West African health Organization’s response to the COVID-19 pandemic in the Economic Community of West African states (ECOWAS) region, 2020 – 2022

**DOI:** 10.1186/s12913-026-14218-6

**Published:** 2026-02-27

**Authors:** Virgil Kuassi Lokossou, Aishat Bukola Usman, Clementine Sorho, Tome CA, William Bosu, Sybil Ossei-Agyeman-Yeboah, Yves Mongbo, Olivier Manigart, Felix Agbla, Issiaka Sombie, Melchior Athanase Aïssi

**Affiliations:** 1 West African Health Organization, Bobo-Dioulasso, Burkina Faso; 2 Ecole de Santé Publique, Université Libre de Bruxelles, Brussels, 1050 Belgium

**Keywords:** Intra-action review, COVID-19, ECOWAS, Regional response, Preparedness, Health security

## Abstract

**Background:**

The West African Health Organization (WAHO) coordinated the regional response to COVID-19 across the 15 ECOWAS Member States. The scale and duration of the pandemic exposed structural weaknesses requiring systematic evaluation. This intra-action review (IAR) assessed WAHO’s performance, operational gaps, and lessons learned from 2020 to 2022 to strengthen future regional preparedness and response.

**Methods:**

A convergent mixed-methods design was used, integrating a targeted desk review, an online survey of WAHO staff, Member State focal points, and liaison officers, 18 key informant interviews, and data from structured IAR meetings conducted between 2021 and 2022. Quantitative data were analyzed descriptively, while qualitative interviews, document, and IAR outputs underwent thematic analysis. Integration was achieved through triangulation, convergence coding, and joint displays across nine core response domains, including coordination, surveillance, laboratory systems, logistics, digital information systems, risk communication, workforce capacity, and cross-border collaboration.

**Results:**

Sixty-six respondents completed the survey (63.5% response rate), and 18 interviews were conducted. The highest-rated domains were coordination, laboratory, vaccination readiness, and cross-border collaboration, while digital information systems scored lowest. Qualitative data highlighted WAHO’s strong political convening role, rapid dissemination of harmonized guidance, effective pooled procurement of diagnostics and PPE, and expansion of laboratory and workforce capacities. However, persistent challenges emerged: fragmented digital reporting systems, variable connectivity, customs-related logistical delays, and uneven access to training across countries. Divergence was observed between quantitative and qualitative data for risk communication—survey scores were moderate, but interviews revealed widespread infodemic pressure. Integrated findings demonstratestrong convergence between quantitative and qualitative data for coordination, laboratory systems, and cross-border collaboration, while systemic gaps particularly in digital systems and logistics, limited operational efficiency.

**Conclusion:**

WAHO played a critical role in coordinating the ECOWAS COVID-19 response, particularly in strengthening regional coordination, laboratory capacity,and cross-border public health functions. Persistent weaknesses in digital surveillance systems, last-mile logistics, and equitable access to training highlight areas requiring sustained investment. Overall,the findings align with broader regional and global evaluations, underscoring the essential role of strong regional institutions in advancing effective and resilient health security in West Africa. Institutionalizing routine IAR/AAR processes, clarifying mandates, and strengthening digital infrastructure and workforce systems are key priorities for enhancing preparedness for future public health emergencies.

**Supplementary information:**

The online version contains supplementary material available at 10.1186/s12913-026-14218-6.

## Background

The COVID-19 pandemic posed unprecedented health, economic, and social challenges globally, with particularly profound implications for regions with fragile health systems including West Africa [1, 2]. The rapid spread of SARS-CoV-2 exposed longstanding structural weaknesses, including limited diagnostic capacity, inadequate surveillance systems, shortages of trained health workers, and constrained supply chains, which hindered timely detection and response across ECOWAS Member States [1, 3, 4]. These systemic challenges underscore the critical need for coordinated regional action to strengthen preparedness, harmonize technical guidance, and ensure efficient mobilization of resources during public health emergencies [5, 6].

In West Africa, the pandemic also led to widespread socio-economic disruptions. Movement restrictions and economic slowdowns exacerbated poverty, reduced remittances, and disproportionately affected populations working in the informal sector, which comprises most of the regional workforce [2, 7]. The health sector faced additional strain from concurrent endemic diseases such as malaria, Lassa fever, and Ebola. In contrast, essential health services were disrupted, consistent with trends observed across sub-Saharan Africa [5, 8]. Inequitable access to vaccines, diagnostics, and personal protective equipment further exacerbated regional disparities due to global supply shortages, delayed procurement, and logistical constraints [5, 7, 9].

Within this context, the West African Health Organization (WAHO), the specialized health institution of ECOWAS, played a central role in coordinating the regional COVID-19 response. Under Article III of its founding protocol, WAHO is mandated to harmonize health policies, support disease prevention and control, and strengthen health systems across ECOWAS Member States through collective action and inter-country cooperation [10]. During the COVID-19 pandemic, WAHO convened political and technical coordination platforms, disseminated harmonized technical guidance, mobilized financial and material resources, facilitated pooled procurement of essential commodities, and supported Member States in surveillance, laboratory strengthening, workforce development, and operational logistics [6, 11].

Despite these contributions, there has been limited systematic documentation evaluating the effectiveness, operational challenges, and lessons learned from WAHO’s regional response to COVID-19. Evidence from other African and global regional bodies has demonstrated that real-time evaluations such as intra-action reviews are essential for identifying strengths, gaps, and opportunities for improvement during prolonged public health emergencies [5, 12]. Similar analyses have emphasized the importance of institutional clarity, regional coordination mechanisms, and adaptive learning to strengthen preparedness and response capacities [13, 14].

To address this evidence gap, the West African Health Organization undertook a formal evaluation of its regional COVID-19 response using the World Health Organization’s Intra-Action Review (IAR) methodology [15]. This study aimed to assess WAHO’s performance, identify operational gaps, and document lessons learned from the regional response to COVID-19 in the ECOWAS region between 2020 and 2022, to inform future preparedness and strengthen regional health security.

## Methods

### Study design

This evaluation employed a convergent mixed-methods design, consistent with WHO’s Intra-Action Review (IAR) approach [9]. Qualitative and quantitative data were collected concurrently, analyzed separately, and merged during interpretation to generate a comprehensive assessment of the West African Health Organization’s (WAHO) COVID-19 response in the ECOWAS region. This design enabled triangulation across data sources and strengthened the credibility and completeness of findings.

The evaluation covered the period February 2021 to May 2022, reflecting the major phases of WAHO’s regional COVID-19 response.

### Study setting and population

The study targeted stakeholders who were directly involved in regional coordination and implementation of COVID-19 response activities. These included:

- WAHO technical officers and departmental leads
- ECOWAS Member State representatives (e.g., National Public Health Institutes, Ministries of Health)
- Implementing partners and technical agencies (WHO AFRO, Africa CDC, GIZ, AfDB, USAID, World Bank)

Participants were eligible if they had direct operational experience with WAHO’s COVID-19 response between January 2020 and May 2022.

### Data sources and data collection procedures

Four main sources of data informed the evaluation:(i) a targeted desk review,(ii) in-depth stakeholder interviews,(iii) an online survey, and(iv) structured IAR meetings.

#### Desk review

A targeted desk review synthesized existing documentation produced by WAHO and partners during the pandemic. Documents included situation reports, meeting minutes, operational guidelines, procurement and logistics reports, mission reports, and donor communications.

The desk review aimed to:

- map WAHO’s COVID-19 response activities and timelines
- identify operational processes and outputs
- inform the development of interview and survey tools
- provide contextual information for triangulation

Documents were screened for relevance and extracted using a structured matrix. Findings were summarized thematically. The review was narrative, not systematic, and served to support the mixed-methods evaluation.

#### In-depth stakeholder interviews (qualitative component)

### Sampling and recruitment

Purposive sampling ensured representation across operational pillars and institutions. Eligible participants were invited via email and provided informed consent.

### Interviewer characteristics and reflexivity

Two trained qualitative researchers with experience in IAR processes conducted the interviews. They had no supervisory relationship with participants, reducing potential bias.

### Interview guide development

A semi-structured interview guide adapted from WHO IAR trigger questions covered(i) governance and coordination, (ii) surveillance and laboratory systems, (iii)logistics and procurement, (iv)digital information systems, (v)risk communication, (vi) workforce capacity, and (vi) cross-border collaboration and partnerships. The guide was pilot tested for clarity.

### Data collection

Interviews were conducted virtually (Zoom/Teams), lasted 45–60 minutes, and were audio-recorded with permission. Interview notes complemented recordings.

### Data management and trustworthiness

Recordings were transcribed verbatim and de-identified. Two analysts independently coded transcripts, compared results, and resolved discrepancies by consensus. Data saturation was reached after 16 interviews; however, 18 interviews were included in the analysis. Member checking was not conducted due to time constraints.

#### Online survey (quantitative component)

### Survey development

A structured questionnaire (52 items) was developed using(i)WHO IAR domains, (ii) WAHO’s COVID-19 response pillars, and (iii) Findings from the desk review and early interviews.

Domains included governance, coordination, surveillance, laboratory systems, logistics, digital systems, workforce capacity, risk communication, and cross-border collaboration. The survey was available in English and French.

The online survey instrument consisted of a structured questionnaire comprising multiple item types. The core of the instrument included Likert-type questions with five response options (1 = Very poor, 2 = Poor, 3 = Fair, 4 = Good, 5 = Excellent) designed to capture respondents’ perceptions of WAHO’s performance across key COVID-19 response domains. In addition, binary and categorical questions were included to collect information on respondent characteristics, institutional affiliation, and level of involvement in COVID-19 response activities. Conceptually related Likert-type items were grouped a priori into domain-specific constructs aligned with the World Health Organization (WHO) Intra-Action Review (IAR) framework and WAHO’s COVID-19 response pillars, including coordination, surveillance, laboratory systems, logistics, digital information systems, workforce capacity, risk communication, and cross-border collaboration. For analysis, individual Likert-type items were first examined descriptively, after which composite domain scores were calculated as the mean of all items within each domain, with higher scores indicating more favorable perceived performance. Binary and categorical variables were summarized using frequencies and proportions. These composite scores were used for descriptive comparison across domains and were not intended to represent psychometrically validated latent constructs.

### Pilot testing and validity considerations

The survey instrument was pilot tested among five WAHO staff members with direct involvement in the COVID-19 response. The pilot test aimed to assess face validity and content validity, focusing on clarity of wording, relevance of items to the WHO Intra-Action Review (IAR) domains, appropriateness of response options, and consistency between the English and French versions. Feedback from the pilot testing informed minor refinements to item phrasing and layout. The survey was designed as a rapid evaluative tool and was not formally assessed for construct validity or internal consistency reliability (e.g., Cronbach’s alpha). Consequently, while conceptually related Likert-type items were grouped into domain-level composite scores for descriptive analysis, these scores do not represent psychometrically validated constructs. Minor adjustments were made. See additional File 3.

### Sampling frame and administration

The sampling frame included WAHO staff involved in COVID-19 activities, Member State liaison officers, and partner agencies. The questionnaire was administered via a secure Google Forms link, with two reminders sent over two weeks.

### Inclusion and exclusion criteria

**Inclusion criteria:** individuals directly involved in the COVID-19 regional response and familiar with WAHO’s activities.

**Exclusion criteria:** individuals without direct knowledge of regional coordination or who did not consent.

### Data handling and protection

No personal identifiers were collected. Data were stored securely in password-protected files.

### Quantitative analysis

Survey data were exported to Excel/STATA and analyzed descriptively (frequencies, proportions, means). Likert-type items were treated as ordinal data and summarised descriptively, while domain-level composite scores were calculated as the mean of related items and used solely for descriptive comparison across domains; no inferential statistical analyses were conducted, and formal assessment of score normality was therefore not performed.

#### Intra-action review (IAR) meetings

In alignment with the WHO IAR methodology, the evaluation incorporated findings from structured IAR meetings conducted between May 2021 and May 2022. These meetings served as an additional qualitative data source, allowing real-time reflection and validation of findings.

##### (i) WAHO Member States focal points meeting (7 May 2021)

This meeting convened Directors of National Public Health Institutes, Ministry of Health representatives, and WAHO Liaison Officers to gather Member State perspectives on WAHO’s COVID-19 support, challenges, and coordination gaps. Discussions were documented using standardized WHO IAR templates.

##### (ii) Pillar-level workshops (March–April 2022)

Ten workshops were held to review response performance across the following pillars(i)Coordination, planning, and monitoring(ii)Risk communication, community engagement, and infodemic management(iii)Surveillance and health information systems(iv)National laboratory systems(v)Operational support and logistics(vi)Essential health services(vii)Capacity building and workforce development(viii)Vaccination(ix)Finance and resource mobilization and (x)Cross-cutting issues (gender, digital health, innovation).

Participants included WAHO staff, ECOWAS Commission representatives, liaison officers, and technical partners. Each workshop applied WHO IAR trigger questions and documentation tools to identify best practices, gaps, and recommendations.

##### (iii) All-pillar validation workshop (5–6 May 2022)

More than 80 participants from WAHO, RCSDC, ECOWAS departments, Member States, and partner agencies (WHO AFRO, Africa CDC, GIZ, AfDB, USAID, World Bank) convened to validate findings from all response pillars. A cross-pillar synthesis session consolidated emerging themes, lessons learned, and recommendations.

### Use of IAR meetings in analysis

Outputs from all IAR meetings, session notes, workshop summaries, and documented recommendations were treated as primary qualitative data. These were integrated into the thematic analysis and triangulated with interview, survey, and desk review findings.

## Data analysis

### Qualitative analysis

Qualitative data were analyzed using an inductive thematic approach. Interview transcripts, desk review matrices, and IAR meeting outputs underwent inductive thematic analysis following a seven-step framework: Familiarization, coding, initial theme development, theme review, theme refinement, naming themes, and narrative synthesis [10]. Transcripts and documents were initially open-coded line by line to generate preliminary codes. These codes were iteratively grouped into higher-order categories, subthemes, and final themes through constant comparison. Themes were mapped to WAHO’s COVID-19 response domains and WHO IAR pillars. A coding tree was developed to document this analytic progression and to enhance transparency and trustworthiness. The final coding tree, illustrating the development from initial codes to themes, is presented in Table 1. Discrepancies in coding were resolved through discussion among the analytic team.

**Table 1:** Qualitative coding tree showing theme development

Level 1: Initial Codes	Level 2: Categories	Level 3: Subthemes	Final Themes
Regional coordination meetings	Governance & coordination	WAHO as a unifying regional actor	Strengthened Regional Coordination and Leadership
Information sharing platforms	Governance & coordination	Rapid convening and information exchange	
Political leadership	Governance & coordination		
PCR procurement	Laboratory & surveillance systems	Diagnostics support	Improvements in Laboratory and Surveillance Capacity
Genomic sequencing training	Laboratory & surveillance systems	Diagnostics support	
Surveillance reporting challenges	Laboratory & surveillance systems	Persistent gaps in digital surveillance	
PPE procurement	Operations & logistics	Effectiveness of procurement mechanisms	Operational and Logistics Support Accelerated Country Response
Customs clearance delays	Operations & logistics	Logistical delays	
Emergency supply distribution	Operations & logistics		
Workforce training	Human resources	Training and capacity building	Workforce Development Enhanced National Capacity
Mentorship programs	Human resources	Training and capacity building	
Unequal access to training	Human resources	Need for more decentralized training	
Cross-border SOPs	Regional collaboration	Harmonized guidance	Cross-Border Collaboration as a Regional Strength
Joint surveillance activities	Regional collaboration	Harmonized guidance	
Sustainability concerns	Regional collaboration	Sustainability concerns	

### Mixed-methods integration analysis

Each data type was analyzed separately:

- Qualitative data were analyzed using inductive and deductive thematic analysis. Codes were developed from the interview guide and emergent patterns in the transcripts. Themes were derived collaboratively by the analysis team.
- Quantitative survey data were analyzed descriptively to characterize respondent perceptions and identify trends across operational domains.
- Desk review findings served as contextual evidence and were used to verify or contrast interview and survey results.

**Fig. 1:**
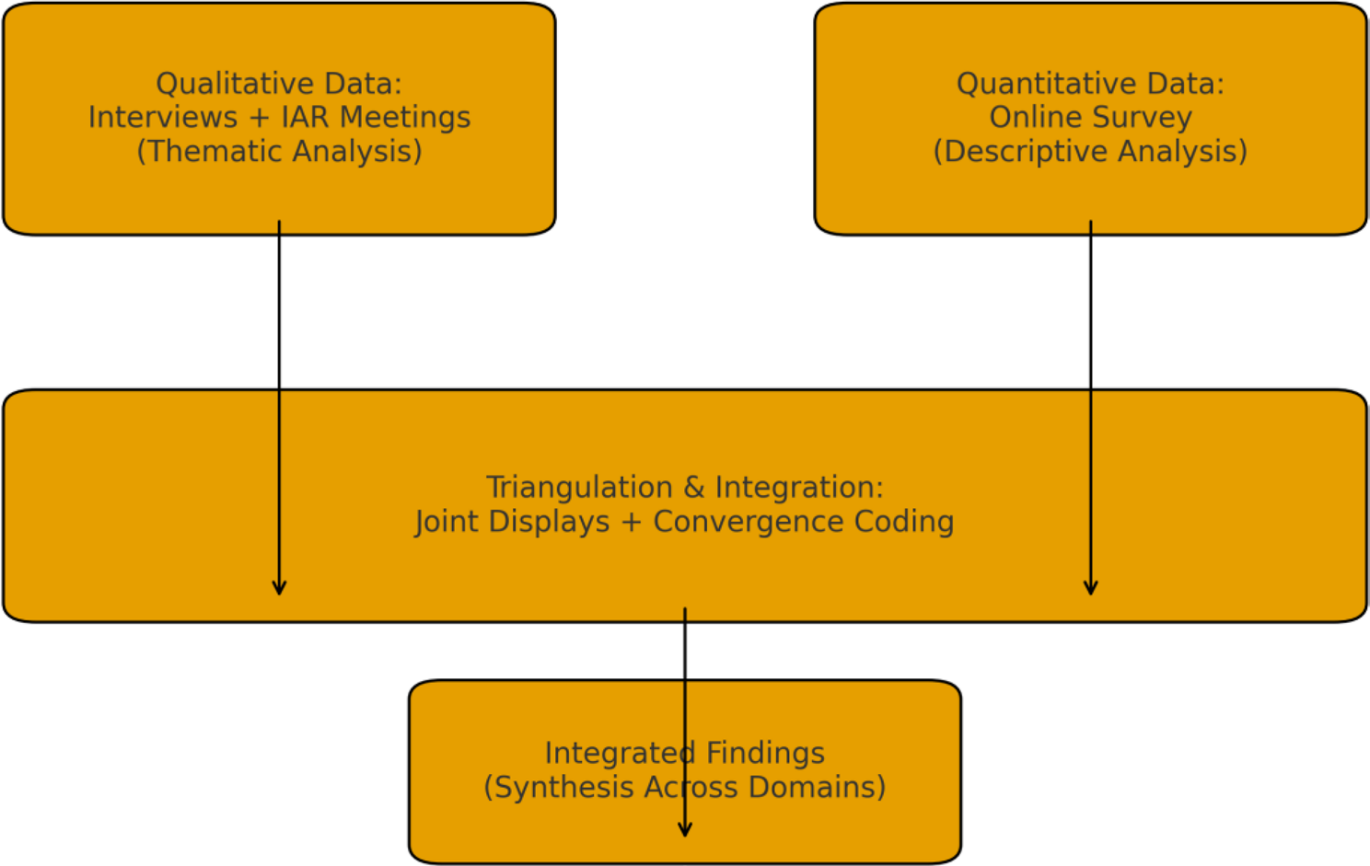
A schematic summary of integration

Integration occurred during the interpretation stage using joint display matrices, convergence coding logic, and triangulation of qualitative and quantitative findings. The integration process compared areas of convergence, complementarity, and divergence across data sources. A schematic summary of the integration approach is provided in Fig. 1.

## Results

A total of 65 respondents completed the online survey out of 102 invited, yielding a 63.5% response rate, and 18 key informants participated in in-depth interviews. In addition, qualitative data were collected through a series of Intra-Action Review (IAR) meetings held between March and April 2022. Findings are presented in three sections: (1) quantitative survey results, (2) qualitative findings, and (3) integrated mixed-methods synthesis.

### Quantitative findings

Survey responses were collected using Likert-type items, and results are presented as domain-level composite scores derived from conceptually related items. As the survey was not subjected to formal psychometric validation, these composite scores should be interpreted as descriptive indicators of perceived performance rather than validated latent constructs.

### Participant characteristics

Among the 66 survey respondents, 42(63.6%) were WAHO technical staff, 16 (24.2%) were ECOWAS Member State focal points, and 8 (12.2%) were liaison officers. Respondents represented all 15 ECOWAS countries.

### Domain-level performance scores

Domain-level performance scores represent descriptive composite summaries derived from multiple Likert-type items; mean scores are reported to aid interpretability and comparison across domains and should not be interpreted as estimates derived from validated or normally distributed scales.

Respondents rated WAHO’s performance across nine domains on a 5-point Likert-type scale (1 = Very Poor to 5 = Excellent). Highest-rated domains: Coordination (mean = 4.1 ± 0.85), cross-border collaboration (mean = 4.0 ± 0.87), and vaccination support (mean = 3.9 ± 0.84). Lowest-rated domain: Digital health and information systems (mean = 3.4 ± 1.06). Domain-level performance scores showed variability in respondent perceptions, with standard deviations ranging from 0.84 (Vaccination Support) to 1.08 (Operational Support & Logistics), indicating greater consensus in some domains than others.

Most respondents (78%) agreed that WAHO provided timely and actionable regional guidance. In addition, 83% rated WAHO’s procurement and distribution of diagnostic supplies as good to excellent. However, 41% of participants reported experiencing delays with digital reporting tools. Furthermore, 72% rated WAHO’s coordination of cross-border surveillance as good to excellent. Overall, these patterns are consistent with regional operational realities observed during COVID-19, particularly the early bottlenecks associated with digital reporting systems.

Domain-level performance scores are presented using both response distributions (n, %) for individual Likert-type items and descriptive composite mean scores derived from related items within each domain. The mean scores with standard deviations are reported to aid interpretability and comparison across domains and should be interpreted as descriptive summaries rather than estimates based on validated or normally distributed scales, as shown in Table 2.

**Table 2:** Domain-level performance scores based on Likert-type items (*n* = 65)

Domain	1 Very Poor n (%)	2 Poor n (%)	3 Fair n (%)	4 Good n (%)	5 Excellent n (%)	Mean score with standard deviation
Coordination & Planning	1 (2%)	2 (3%)	9 (14%)	36 (55%)	17 (26%)	4.1 ± 0.85
Surveillance & Health Information	3 (5%)	5 (7%)	17 (26%)	30 (45%)	11 (17%)	3.7 ± 0.99
Laboratory Systems	1 (2%)	3 (5%)	17 (26%)	32 (48%)	13 (19%)	3.9 ± 0.91
Operational Support & Logistics	5 (7%)	5 (7%)	19 (29%)	25 (38%)	12 (19%)	3.6 ± 1.08
Workforce & Capacity Building	3 (5%)	3 (5%)	17 (26%)	30 (45%)	13 (19%)	3.8 ± 0.98
Digital Health & Information Systems	7 (10%)	6 (9%)	22 (33%)	25 (38%)	6 (10%)	3.4 ± 1.06
Risk Communication & Community Engagement	5 (7%)	5 (7%)	22 (33%)	25 (38%)	9 (14%)	3.5 ± 1.04
Vaccination Support	1 (2%)	1 (2%)	17 (26%)	34 (52%)	13 (19%)	3.9 ± 0.84
Cross-Border Collaboration	1 (2%)	3 (5%)	11 (17%)	33 (50%)	17 (26%)	4.0 ± 0.87

**Note***: Percentages may not sum to 100 due to rounding. Domain-level scores were calculated as the mean of conceptually related Likert-type items (5-point response options). Standard deviations (SDs) were derived from the response distributions to indicate variability in perceptions. Mean scores and SDs are presented for descriptive purposes only to summarise perceived performance across domains. No assumptions of normality were made, and domain constructs were not psychometrically validated*

#### Qualitative findings

**Fig. 2:**
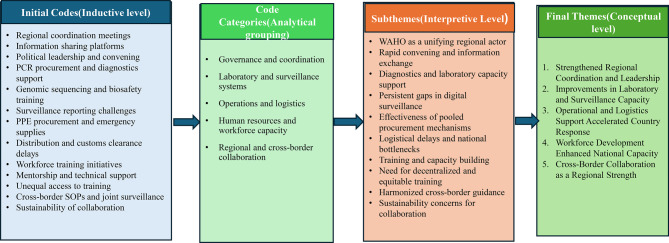
Coding tree illustrating development of qualitative themes

Qualitative data from 18 interviews and three IAR meeting streams yielded five major themes. Qualitative findings were derived from inductive thematic analysis of key informant interviews and Intra-Action Review (IAR) discussions. Initial codes were generated from repeated patterns in the data and subsequently organized into analytical categories, subthemes, and overarching themes through iterative team discussions. The analytic progression from initial codes to final themes is illustrated in Fig. 2, which demonstrates the transparency and rigor of the coding process. Five major themes emerged, reflecting both strengths and persistent challenges in WAHO’s regional coordination and support during the COVID-19 response. Themes were refined until analytic saturation was reached, and discrepancies were resolved through consensus.

## Theme 1: strengthened regional coordination and leadership

As illustrated in Fig. 2, this theme emerged from codes related to regional coordination meetings, information-sharing mechanisms, and political convening, reflecting WAHO’s role as a unifying regional actor during the COVID-19 response. Participants consistently described regional coordination and leadership as a foundational strength of the response, particularly highlighting the role of WAHO as a unifying regional actor. Respondents emphasized that WAHO’s leadership enhanced coherence across Member States, reducing fragmentation and enabling collective action during a period of uncertainty. This coordination role was perceived as especially critical in aligning national responses and ensuring the timely sharing of information across the region.

As one Member State focal point explained:

Without WAHO, each country would have acted alone. They aligned us and kept us informed. *(Member State Focal Point 07)*

In addition to institutional alignment, participants valued the rapid convening of coordination platforms, particularly during the early phase of the pandemic. Regular meetings created predictable channels for information exchange, enabling countries to share challenges, lessons learned, and evolving response strategies in real time.

Weekly calls created a rhythm. We always knew what others were facing. *(WAHO Staff 04)*

These findings illustrate how leadership and coordination mechanisms functioned not only as governance structures but as practical tools that shaped response efficiency and collective problem-solving.

## Theme 2: improvements in laboratory and surveillance capacity

Derived from codes concerning diagnostic procurement, laboratory training, and surveillance reporting challenges (Fig. 2), this theme captures both the expansion of laboratory capacity and persistent gaps in digital surveillance systems. Participants described notable improvements in laboratory capacity, particularly through regional procurement and diagnostic support. Respondents frequently highlighted WAHO’s role in facilitating access to PCR machines and test kits, which enabled countries to scale up testing capacity during critical phases of the response.

The region could not have tested at scale without the support from WAHO. *(Partner Agency 02)*

However, while laboratory capacity improved, participants identified persistent weaknesses in digital surveillance systems. Despite the availability of reporting tools, challenges related to connectivity, training, and uneven system adoption limited the effective use of surveillance data. These gaps were perceived to undermine real-time situational awareness and data-driven decision-making.

The tools were there, but connectivity and training were uneven. *(Member State Representative 06)*

Together, these findings suggest that while diagnostic capacity benefited from coordinated regional investments, digital surveillance remained a structural bottleneck that constrained the full utilisation of improved laboratory systems.

## Theme 3: operational and logistics support accelerated country response

As shown in Fig. 2, this theme developed from operational codes related to pooled procurement, emergency supply distribution, and customs clearance processes, highlighting both efficiencies and delays in logistics support.

Operational and logistics support emerged as a critical enabler of national response activities. Participants consistently described WAHO’s pooled procurement mechanisms as instrumental in securing essential supplies at a time of global shortages and inflated prices. This approach was perceived to enhance equity and cost-efficiency across Member States.

The pooled procurement mechanism saved us—prices were impossible on the open market. *(Member State Representative 10)*

Despite these strengths, respondents acknowledged that logistical challenges persisted, particularly related to transport, customs clearance, and distribution timelines. These delays occasionally limited the timeliness of response activities at the country level.

Sometimes the commodities arrived weeks later due to customs procedures. *(Member State Representative 05)*

These findings highlight a dual narrative of operational effectiveness tempered by structural constraints beyond the direct control of regional coordination mechanisms.

## Theme 4: workforce development enhanced national capacity

This theme, mapped in Fig. 2, emerged from codes associated with workforce training, mentorship, and capacity-building initiatives, alongside concerns about unequal access to training across Member States. Participants consistently identified workforce development as a key contributor to strengthened national response capacity. Training initiatives, particularly in specialised technical areas such as genomic sequencing and biosafety, were perceived to address critical skill gaps exposed during the pandemic.

The trainings on sequencing and biosafety filled the gaps we had before COVID. *(Laboratory Specialist 01)*

However, participants also noted inequities in access to training opportunities across Member States. Some respondents perceived that training activities were initially concentrated in certain linguistic or geographic groups, limiting broader regional impact.

Francophone countries got more sessions initially. Others felt left behind. *(NPHI Director 05)*

These findings underscore the importance of equitable and decentralised capacity-building strategies to ensure sustained regional preparedness.

## Theme 5: cross-border collaboration as a regional strength

Grounded in codes related to harmonized cross-border protocols, joint surveillance, and sustainability concerns (Fig. 2), this theme reflects the perceived value and fragility of regional cross-border collaboration mechanisms. Cross-border collaboration was widely perceived as a significant strength of the regional response. Participants highlighted the role of harmonised guidance and standard operating procedures in facilitating safe border reopening and coordinated public health actions among neighbouring countries.

WAHO’s cross-border SOPs allowed us to reopen safely with our neighbors. *(NPHI Director 01)*

At the same time, respondents expressed concerns about the sustainability of these gains. Participants emphasised that improvements in cross-border collaboration were closely tied to emergency funding and political attention, raising questions about long-term institutionalisation.

Collaboration improved during COVID, but we worry it won’t last without funding. *(Partner Agency 04)*

These findings suggest that while cross-border coordination mechanisms proved effective during the emergency, sustained investment is required to embed them into routine regional health security frameworks.

Overall, integration demonstrated that quantitative domain scores captured perceived levels of performance, while qualitative findings elucidated the institutional, operational, and contextual factors shaping those perceptions. This mixed-methods approach strengthened interpretation by revealing not only performance levels but also the mechanisms and constraints influencing regional public health response effectiveness

### Mixed-methods integration

Integration of quantitative and qualitative findings demonstrated meaningful patterns of convergence, complementarity, and divergence across response domains, highlighting the added value of a mixed-methods approach in understanding regional public health system performance.

### Convergent findings

Strong convergence was observed in domains where quantitative performance ratings aligned closely with qualitative experiences. Coordination and Planning (mean = 4.1 ± 0.85) and Cross-Border Collaboration (mean = 4.0 ± 0.87) both demonstrated high survey scores, which were corroborated by qualitative findings describing WAHO’s role as a unifying regional convener, the effectiveness of rapid coordination mechanisms, and the value of harmonised cross-border guidance. Participant narratives illustrated how these coordination structures translated into practical benefits, explaining the consistently high-performance ratings (Table 3).

**Table 3:** Convergence between survey and qualitative data

Domain	Survey finding (n = 65)	Qualitative finding	Integration conclusion
Coordination	**High performance (mean = 4.1 ± 0.85)**, indicating strong perceived effectiveness of coordination mechanisms	WAHO perceived as a strong regional convener facilitating alignment across Member States	Convergence
Laboratory systems	**High performance (mean = 3.9 ± 0.99)**, reflecting adequate diagnostic and laboratory support	Strong support for procurement of supplies and training	Convergence
Digital systems	**Low performance (mean = 3.4 ± 1.06)**, indicating challenges in digital health and information systems	Recurrent complaints about fragmented tools and limited interoperability	Convergence
Logistics	**Moderate performance (mean = 3.6 ± 1.08)**, suggesting mixed perceptions of operational efficiency	Mixed praise for pooled procurement offset by concerns about logistical delays	Complementarity
Cross-border collaboration	**High performance (mean = 4.0 ± 0.87)**, reflecting effective cross-border coordination	Harmonised SOPs for border reopening validated by interviews	Convergence
Workforce	**Moderate-to-high performance (mean = 3.8 ± 0.98)**, indicating strengthened capacity with remaining gaps	Training is appreciated but uneven across Member States	Complementarity

**Note:***Survey scores were derived from 5-point Likert-type items (1 = very poor to 5 = excellent). For interpretive purposes, mean scores (presented with standard deviations) were categorised as follows: low performance (<3.5), moderate performance (3.5–3.9), and high performance (≥4.0). These categories are descriptive and intended to support integration with qualitative findings*

Similarly, Digital Health and Information Systems, which received the lowest quantitative score (mean = 3.4 ± 1.06), showed convergence with qualitative findings that consistently highlighted fragmented digital platforms, uneven connectivity, and limited interoperability. The low standard deviation for Coordination (SD = 0.85) suggests strong agreement on WAHO’s convening role, whereas higher variability in Digital Systems (SD = 1.06) reflects inconsistent user experiences and structural inequities (Table 3). This alignment suggests that respondents’ lower ratings accurately reflected persistent structural challenges in digital systems.

To aid interpretation, survey mean scores presented with standard deviations were categorised into low, moderate, and high-performance levels and examined alongside qualitative findings to assess convergence, complementarity, or divergence across domains

### Complementary findings

Complementarity was evident in domains where quantitative scores captured overall performance levels, while qualitative data provided deeper insight into the underlying drivers of those scores. For Operational Support and Logistics (mean = 3.6 ± 1.08), the survey findings suggested moderate performance. In contrast, qualitative narratives revealed a nuanced balance between effective pooled procurement mechanisms and persistent delays related to transportation and customs procedures. Qualitative data thus contextualised the moderate score by revealing offsetting strengths and constraints.

A similar complementary pattern was observed for Workforce and Capacity Building (mean = 3.8 ± 0.98). While survey results indicated relatively strong capacity, qualitative findings highlighted inequities in access to training and the need for more decentralised capacity-building approaches. These insights help explain why the domain performed well overall yet remained an area requiring further investment.

### Divergent findings

Divergence was most apparent in the Risk Communication and Community Engagement domain. Although survey respondents rated this area as moderate (mean = 3.5 ± 1.04), qualitative findings revealed more substantial challenges related to misinformation, infodemics, and community mistrust. The high standard deviation (SD = 1.04) further suggests polarized perceptions that the survey mean alone masked. This divergence suggests that structured survey items may have captured formal communication activities, while interviews illuminated contextual and behavioural challenges that were less visible in quantitative ratings. The qualitative findings, therefore, expanded the understanding of the domain by uncovering gaps not fully reflected in survey scores.

### Value of integration

Overall, integration demonstrated that quantitative findings provided a broad assessment of perceived performance, while qualitative data offered critical contextual explanations, illuminated system dynamics, and identified areas where survey ratings alone were insufficient. By examining convergence, complementarity, and divergence, this study illustrates how mixed-methods approaches can strengthen interpretation, enhance validity, and support more nuanced policy-relevant conclusions regarding regional public health preparedness and response.

## Discussion

This mixed-methods evaluation provides a comprehensive understanding of the West African Health Organization’s (WAHO) role in coordinating and supporting the ECOWAS regional response to COVID-19. By integrating evidence from surveys, key informant interviews, and Intra-Action Review (IAR) meetings, the study identifies both the strengths and persistent gaps in regional preparedness and response functions. These findings align with and add nuance to existing literature on regional public health coordination in Africa and globally.

## Regional coordination as a core strength

The findings highlight regional coordination and planning as a central enabling factor in the ECOWAS COVID-19 response, underscoring WAHO’s role as a trusted regional convener. This is consistent with findings from Africa CDC and WHO analyses, which emphasize that effective regional coordination was one of the defining strengths of Africa’s COVID-19 response [11–13]. Like evaluations of the European CDC and the Pan-American Health Organization, timely regional guidance reduced fragmentation and facilitated coherent action across borders [14, 15]. Our findings demonstrate that WAHO played a comparable role in West Africa, reinforcing the value of regional institutions in collective health security.

## Laboratory strengthening and surveillance gains reflect continental trends

The observed improvements in laboratory support on PCR capacity, genomic sequencing, and biosafety training across Africa reflect broader continental trends documented during the COVID-19 response [12, 16]. The effectiveness of pooled procurement for diagnostics mirrors mechanisms described in global analyses of supply chain innovations during the pandemic [17]. However, the moderate rating for surveillance systems and qualitative concerns about digital reporting bottlenecks align with studies showing that digital health infrastructure in many African countries lagged laboratory expansion [18, 19]. These challenges have been well established in WHO assessments and are not unique to West Africa.

## Operational and logistics challenges reflect systemic constraints

The mixed perceptions of logistics performance in this evaluation mirror patterns documented in other African regions, where supply chains were hindered by global market inequities, border closures, and customs delays [17, 20]. While WAHO’s pooled procurement was widely appreciated, the delays reported by interview participants are consistent with Africa CDC and World Bank analyses showing that regional bodies often lacked leverage over national customs processes during the pandemic [13, 21]. This evaluation’s findings, therefore, corroborate evidence that logistical challenges in global emergencies are often structural rather than institution-specific.

## Workforce development improved capacity, but gaps remain

Workforce development emerged as an important contributor to national response capacity in Africa’s COVID-19 response [16, 22]. Interview feedback regarding unequal access to training echoes findings from ECOWAS and WHO evaluations, noting linguistic, connectivity, and resource disparities across Member States [23]. These inequities point to the need for more decentralized and digitally enabled training approaches, consistent with global recommendations for resilient workforce systems [24].

## Digital health remains a regional weakness

Digital health and information systems emerged as a persistent regional challenge, consistent with evidence that digital transformation remains the weakest component of health security systems across Africa [19, 25]. Studies from the WHO, Africa CDC, and the BMJ Global Health have consistently documented challenges related to interoperability, connectivity, and training gaps, which are echoed by our interview and IAR findings [18, 25]. The divergence between survey ratings and qualitative descriptions of severe infodemic pressure reflects what several authors have described as the complexity of risk communication, where quantitative tools often undercapture the intensity and spread of misinformation [26].

## Cross-border collaboration as a recognized regional asset

Cross-border collaboration was one of the highest-performing domains, which aligns with pre-existing literature demonstrating that WAHO and ECOWAS have historically strong structures for cross-border health security [27, 28]. Our findings reaffirm this strength, reflecting evidence from evaluations of Ebola, Lassa fever, and meningitis responses where harmonized protocols and joint surveillance improved early detection and response across borders [27, 29].

## Implications for policy and practice

The findings reinforce the argument that regional institutions are indispensable for managing cross-border health threats, particularly in regions with porous borders such as West Africa. Strengthening domestic financing for regional health security aligns with WHO and African Union recommendations calling for sustainable financing of regional entities [13]. The persistent weaknesses in digital systems underscore the urgent need for investment in digital surveillance, data governance, and capacity building, which are core priorities in the Global Health Security Agenda and Africa CDC’s New Public Health Order [25]. Workforce development should be expanded through decentralized, context-adapted approaches, consistent with lessons from prior epidemics [22, 24]. Finally, the strong performance of cross-border collaboration highlights the importance of institutionalizing these mechanisms within national and regional preparedness plans.

## Strengths and limitations

A key strength of this study is its convergent mixed-methods design, which allowed triangulation across multiple data sources, including IAR meetings that functioned as real-time validation spaces. Similar methodologies have been recommended in global health emergency evaluations to enhance credibility and trustworthiness [30]. However, some limitations exist. Self-reported survey data may carry biases, and virtual interviews may have limited depth for certain participants. Additionally, while descriptive quantitative scores were generated to support integration, these findings align with well-established patterns documented in peer-reviewed literature. (Table 4)

**Table4:** Recommendations

No.	Finding	Evidence from Results	Recommendation
1	Moderate risk communication, with significant infodemic challenges	Survey respondents rated risk communication moderately high (3.5/5), while interviews highlighted widespread misinformation and rumors	Develop context-specific risk communication strategies Train public health staff on infodemic management and crisis communication Implement real-time monitoring tools to detect and address misinformation
2	Generally strong cross-border collaboration with sustainability and standardization gaps	Some countries reported strong coordination; others faced challenges in joint surveillance and response	Standardize cross-border data sharing and response protocols Conduct joint simulation exercises and after-action reviews Establish regional platforms for rapid exchange of surveillance and laboratory data
3	Variability in national-level governance and multisectoral engagement	Differences in task force effectiveness and coordination across countries	Strengthen national epidemic preparedness taskforces with multisectoral representation Align national health security plans with regional frameworks Engage community stakeholders to enhance trust and compliance
4	Resource constraints and reliance on donor support	Interviews highlighted operational challenges and limited domestic funding	Advocate for sustainable domestic financing for epidemic preparedness Facilitate regional resource-sharing for critical interventions Invest in digital tools for reporting, logistics, and communication
5	Workforce gaps in outbreak response	Interview data emphasized limited technical capacity and the need for mentorship	Provide targeted training in outbreak investigation, rapid response, and data analysis Establish regional mentorship programs Encourage continuous professional development aligned with regional health security priorities
6	Need for continuous monitoring and adaptation	Variability in country performance and infodemic challenges	Implement structured monitoring and evaluation systems Use joint assessments and after-action reviews to iteratively improve response protocols Disseminate best practices regionally

## Conclusion

WAHO played a central role in strengthening regional preparedness and emergency response during COVID-19, particularly in coordination, laboratory systems support, and cross-border public health actions. However, persistent challenges related to digital systems, logistics, and equitable access to training highlight areas requiring sustained investment and institutional strengthening. Overall, these findings align closely with regional and global evaluations, underscoring the critical role of strong regional institutions in advancing effective and resilient health security in West Africa.

## Electronic supplementary material

Below is the link to the electronic supplementary material.

Supplementary Material 1

Supplementary Material 2

Supplementary Material 3

## Data Availability

All the data generated or analyzed during this study are included in this published article and its supplementary information files.

## Abbreviations

COVID-19: Coronavirus disease 2019
ECOWAS: Economic Community of West African States
EHS: Essential Health Services
IDP: Internally Displaced Persons
IDSR: Integrated Disease Surveillance and Response
PHEM: Public Health Emergency Management
RCCE: Risk Communication and Community Engagement
RCSDC: ECOWAS Regional Center for Surveillance and Disease Control
