# Supplementary material for: Intra-action review of West African health Organization’s response to the COVID-19 pandemic in the Economic Community of West African states (ECOWAS) region, 2020 – 2022
Source: BMC Health Serv Res. 2026 Feb 27;26:462. doi: 10.1186/s12913-026-14218-6 (PMC13049954; doi:10.1186/s12913-026-14218-6)
Supplement: Supplementary file 1 — Supplementary Material 1 [file 12913_2026_14218_MOESM1_ESM.pdf]

Online Survey Questionnaire

Introduction

You are invited to participate in this survey, which is part of a regional assessment of WAHO's support for the 2024–2025 Mpox response across ECOWAS Member States. The purpose of this questionnaire is to gather your insights on governance, coordination, surveillance, laboratory systems, logistics, workforce capacity, digital tools, risk communication, and cross-border collaboration during the response. Your feedback will help identify strengths, gaps, and lessons learned, and inform future improvements in regional epidemic preparedness and response.

Participation is voluntary, and there are no right or wrong answers. Your responses will remain confidential and will be used only for this assessment.

Consent

By completing this questionnaire, you indicate that you:

1. Understand the purpose of the survey and how the information will be used.
2. Agree to participate voluntarily.
3. Consent to the use of your anonymized responses for analysis, reporting, and dissemination.

If you do not wish to participate, you may exit the survey at any time.

1. Consent to participate:

- Yes
- No

Section A: Respondent Profile

2. Institution/affiliation:

3. Role/function:

4. Country:

5. Years of experience:

6. Level of involvement in COVID-19 response:

- 1
- 2
- 3
- 4
- 5

Section B: Governance & Coordination

7. WAHO provided clear leadership during the pandemic:

- 1 – Strongly Disagree
- 2 – Disagree
- 3 – Neutral
- 4 – Agree
- 5 – Strongly Agree

8. Guidance from WAHO was timely:

- 1 – Strongly Disagree
- 2 – Disagree
- 3 – Neutral
- 4 – Agree
- 5 – Strongly Agree

9. Technical meetings improved coordination:

- 1 – Strongly Disagree
- 2 – Disagree
- 3 – Neutral
- 4 – Agree
- 5 – Strongly Agree

10. Roles of WAHO and partners were clearly understood:

- 1 – Strongly Disagree
- 2 – Disagree
- 3 – Neutral
- 4 – Agree
- 5 – Strongly Agree

11. Coordination platforms were effective:

- Yes
- No

- Comment: ____________________

Section C: Surveillance

12. Regional surveillance guidelines were useful:

- 1 – Strongly Disagree
- 2 – Disagree
- 3 – Neutral
- 4 – Agree
- 5 – Strongly Agree

13. Data reporting was timely:

- 1 – Strongly Disagree
- 2 – Disagree
- 3 – Neutral
- 4 – Agree
- 5 – Strongly Agree

14. Data quality was adequate:

- 1 – Strongly Disagree
- 2 – Disagree
- 3 – Neutral
- 4 – Agree
- 5 – Strongly Agree

15. Integration across Member States was improved:

- 1 – Strongly Disagree
- 2 – Disagree
- 3 – Neutral
- 4 – Agree
- 5 – Strongly Agree

16. Key surveillance gaps:

- Open text response

Section D: Laboratory Systems

17. Diagnostic support from WAHO was adequate:

- 1 – Strongly Disagree

- 2 – Disagree
- 3 – Neutral
- 4 – Agree
- 5 – Strongly Agree

18. Regional genomic sequencing improved variant detection:

- 1 – Strongly Disagree
- 2 – Disagree
- 3 – Neutral
- 4 – Agree
- 5 – Strongly Agree

19. Equipment and supplies were sufficient:

- 1 – Strongly Disagree
- 2 – Disagree
- 3 – Neutral
- 4 – Agree
- 5 – Strongly Agree

20. Training improved laboratory performance:

- 1 – Strongly Disagree
- 2 – Disagree
- 3 – Neutral
- 4 – Agree
- 5 – Strongly Agree

21. Main laboratory challenges:

- Open text response

Section E: Logistics and Procurement

22. Pooled procurement improved availability of supplies:

- 1 – Strongly Disagree
- 2 – Disagree
- 3 – Neutral
- 4 – Agree
- 5 – Strongly Agree

23. Logistics coordination was efficient:

- 1 – Strongly Disagree
- 2 – Disagree
- 3 – Neutral
- 4 – Agree
- 5 – Strongly Agree

24. Supplies arrived on time:

- 1 – Strongly Disagree
- 2 – Disagree
- 3 – Neutral
- 4 – Agree
- 5 – Strongly Agree

25. In-country distribution was effective:

- 1 – Strongly Disagree
- 2 – Disagree
- 3 – Neutral
- 4 – Agree
- 5 – Strongly Agree

26. Main logistics bottlenecks:

- Open text response

Section F: Workforce Capacity

27. Workforce capacity met response needs:

- 1 – Strongly Disagree
- 2 – Disagree
- 3 – Neutral
- 4 – Agree
- 5 – Strongly Agree

28. Virtual training platforms were effective:

- 1 – Strongly Disagree
- 2 – Disagree
- 3 – Neutral
- 4 – Agree
- 5 – Strongly Agree

29. Technical support from WAHO was sufficient:

- 1 – Strongly Disagree
- 2 – Disagree
- 3 – Neutral
- 4 – Agree
- 5 – Strongly Agree

30. Surge capacity was adequate:

- 1 – Strongly Disagree
- 2 – Disagree
- 3 – Neutral
- 4 – Agree
- 5 – Strongly Agree

31. Priority workforce gaps:

- Open text response

Section G: Digital & Data Systems

32. Digital platforms supported effective decision-making:

- 1 – Strongly Disagree
- 2 – Disagree
- 3 – Neutral
- 4 – Agree
- 5 – Strongly Agree

33. Interoperability across systems improved:

- 1 – Strongly Disagree
- 2 – Disagree
- 3 – Neutral
- 4 – Agree
- 5 – Strongly Agree

34. Data dashboards were reliable:

- 1 – Strongly Disagree
- 2 – Disagree
- 3 – Neutral
- 4 – Agree
- 5 – Strongly Agree

35. Data sharing processes were clear:

- 1 – Strongly Disagree
- 2 – Disagree
- 3 – Neutral
- 4 – Agree
- 5 – Strongly Agree

36. Main information system challenges:

- Open text response

Section H: Risk Communication

37. RCCE materials from WAHO were useful:

- 1 – Strongly Disagree
- 2 – Disagree
- 3 – Neutral
- 4 – Agree
- 5 – Strongly Agree

38. Messaging was consistent across countries:

- 1 – Strongly Disagree
- 2 – Disagree
- 3 – Neutral
- 4 – Agree
- 5 – Strongly Agree

39. WAHO provided adequate communication support:

- 1 – Strongly Disagree
- 2 – Disagree
- 3 – Neutral
- 4 – Agree
- 5 – Strongly Agree

40. Social listening mechanisms were used effectively:

- 1 – Strongly Disagree
- 2 – Disagree
- 3 – Neutral
- 4 – Agree
- 5 – Strongly Agree

41. Main RCCE gaps:

- Open text response

Section I: Cross-Border Collaboration

42. Cross-border alerts were timely:

- 1 – Strongly Disagree
- 2 – Disagree
- 3 – Neutral
- 4 – Agree
- 5 – Strongly Agree

43. Border-level coordination was strengthened:

- 1 – Strongly Disagree
- 2 – Disagree
- 3 – Neutral
- 4 – Agree
- 5 – Strongly Agree

44. WAHO support improved movement-related protocols:

- 1 – Strongly Disagree
- 2 – Disagree
- 3 – Neutral
- 4 – Agree
- 5 – Strongly Agree

45. Key cross-border challenges:

- Open text response

Section J: Overall Assessment

4.6 Overall performance of WAHO:

- 1 – Strongly Disagree
- 2 – Disagree
- 3 – Neutral
- 4 – Agree
- 5 – Strongly Agree

47. Greatest strengths:

- Open text response

48. Greatest weaknesses:

- Open text response

49. Most important lessons:

- Open text response

50. Recommendations for improvement:

- Open text response

51. Should WAHO institutionalize routine IAR/AAR?

- Yes
- No

52. Additional comments:

- Open text response
