# Supplementary material for: Intra-action review of West African health Organization’s response to the COVID-19 pandemic in the Economic Community of West African states (ECOWAS) region, 2020 – 2022
Source: BMC Health Serv Res. 2026 Feb 27;26:462. doi: 10.1186/s12913-026-14218-6 (PMC13049954; doi:10.1186/s12913-026-14218-6)
Supplement: Supplementary file 2 — Supplementary Material 2 [file 12913_2026_14218_MOESM2_ESM.pdf]

Annexes

Annex A. List of external in-depth interview participants

The following matrix outlines the list of external stakeholders who took part in the in-depth interviews, moderated by TBI. In total, data collection was undertaken with seven of the fifteen ECOWAS Member States, and nine of ten WAHO partners.

**Member States**	**Partners**
**Burkina Faso**	**ACDC**
**Cape Verde**	**ALCO**
**Cote d'Ivoire**	**ECOWAS**
**Guinea**	**GIZ**
**Nigeria**	**KFW**
**Sierra Leone**	**Netherland Cooperation**
**Togo**	**USAID**
Benin	**WHO / AFRO**
Ghana	**World Bank**
Guinea Bissau	African Development Bank
Liberia	
Mali	
Niger	
Senegal	
The Gambia	

NOTE: The bolded names are those with whom TBI conducted IDIs. The greyed names are those who were unavailable.

Annex B. In-depth interview guides

WAHO IAR: Partner IDI Guide

Introduction to interviewees

Hello and thank you for agreeing to speak to us today.

I am part of an independent team at the Tony Blair Institute for Global Change which is supporting the West African Health Organisation (WAHO) to conduct an internal intra-action review of its support to Member States during the COVID-19 response.

The purpose of this review is to understand WAHO's contribution in supporting a regional pandemic response, and to identify best practices, challenges, and lessons that have been learnt along the way.

The findings from this study will be shared with the WAHO team and other stakeholders including the Bill and Melinda Gates Foundation. They will be used to inform the next phase of WAHO's support to the COVID-19 response and its capacity strengthening priorities.

All your answers are confidential, and you are free not to answer any questions if you do not want to.

The interview should take up to 1 hour. We will start by discussing your organisation's collaboration with WAHO, followed by your perceptions of the role WAHO has played in the pandemic response, its coordination and collaboration with other partners, and finally, your recommendations going forward. Given time constraints, we would appreciate if you could make your answers as succinct as possible.

With your permission I would like to record the interview. The recording will be used by the review team only and will be deleted once the review is complete.

Background – 5 min.

We will start the interview with a couple of a background questions on you and your organisation's relationship and engagement with WAHO.

Q1. Could you please briefly describe the role your organisation played in West Africa's COVID-19 response, vis-à-vis the landscape of other actors who were involved?

- Who were the main actors you collaborated with – development partners, donors, private and/or other sectors, etc.?

Q2. Could you please provide some background information on your organisation's engagement and collaboration with WAHO, in previous years and currently during the COVID-19 response?

- Since when/for how long has your organisation been working with WAHO? As part of long-term engagement or just for the COVID-19 response?
- In what capacity? (e.g. financial, technical (lab, surveillance), strategic; embedded, in an advisory role?)
- How regularly does your organisation engage with WAHO currently?
- Is your organisation supporting the pandemic response at other levels (e.g. country, continental) as well?

Q3. (optional) **What is your specific experience working with WAHO within your organisation?**

- Since when/for how long have you personally been working with WAHO? As part of long-term engagement or just for the COVID-19 response?
- In what capacity? (e.g. financial, technical, strategic; embedded, in an advisory role?)

- How regularly have you engaged with WAHO during the COVID-19 response? Who do you typically engage with?

Role of WAHO – 20 min.

Q4. How would you describe WAHO's role in guiding or implementing the regional COVID-19 response?

- Vis-à-vis other international actors?
- Regional leadership; communication, collaboration, coordination with Member States; communication, collaboration, and coordination with partner organisations; technical support vs. advocacy?
- At the beginning of the pandemic vs. now?

Q5. To what extent do you feel that WAHO's mission and mandate in the pandemic response is well understood?

- By Member States, partners / donors, other regional bodies?
- Is it well understood now?

Q6. How would you describe WAHO's capacity to implement against its role or mandate in general?

- Technically, management, strategy, resourcing, leadership, etc.
- Any change from before the COVID-19 pandemic response, until now?
- What are the most important gaps?

Q7. *(If the partner provided financial/technical/capacity support to WAHO)* **What has been your experience providing capacity support to WAHO? Do you feel it was/has been effective – why, why not?**

- Was WAHO able to clearly articulate its needs and key areas for support?

Q8. Where in particular has WAHO's support added value to your ongoing efforts in the pandemic response?

- *Probe, if needed:* Were there aspects of your country's COVID-19 response that would not have been possible or that would have been more challenging without the support from WAHO (e.g. access to PPE, staff deployment, training, etc.)?

Q9. Overall, what could have been improved about WAHO's support in the COVID-19 response?

Communication, Coordination and Collaboration with Partners – 20 min.

Q10. How effectively do you think WAHO was able to improve the coordination of international support to West Africa's COVID-19 response? Please explain.

Q11. How can WAHO improve coordination, communication and collaboration with other partners operating across West Africa?

- *Probe, if needed*: To support in the ongoing COVID-19 response and to support in the medium- and longer-term

Recommendations – 15 min.

Q12. What recommendations do you have for WAHO, in terms of supporting the ongoing COVID-19 response and recovery and/or in improving Member States' preparedness and resilience to future pandemics?

- *Probe* on:
  - Local capacity building (support to Member States' health systems strengthening)
  - Further WAHO integration (structural)
  - Strengthening WAHO capacity to better fulfil its mandate (e.g. improving skills, leadership, systems, structures, delivery mechanisms, etc.)
  - Other?

Q13. In one word, what do you think is the most important challenge for the West Africa region going forward in responding to COVID-19? Why?

Q14. What would you say the key lessons are from the COVID-19 response regarding:

- How best to support Member States / NPHIs both during COVID and in preparation for future health emergencies?
- How best to coordinate multiple actors to ensure synergies and additionality?
  - Structures / systems; ensuring resilience; sustainability of capacity / support

WAHO IAR: Member States IDI Guide

Introduction to interviewees

Hello and thank you for agreeing to speak to us today.

I am part of an independent team at the Tony Blair Institute for Global Change which is supporting the West African Health Organisation (WAHO) to conduct an internal intra-action review of its support to member states during the COVID-19 response.

The purpose of this review is to share understanding of WAHO's contribution in support of your country's COVID-19 pandemic response to identify best practices, challenges, and lessons that have been learnt along the way.

As part of this review, we are also interested to understand the perspectives of ECOWAS member states on WAHO's contribution to the pandemic response so far. The findings from this study will be shared with the WAHO team and other stakeholders including the Bill and Melinda Gates Foundation, who are funding the review.

All your answers are confidential, and you are free not to answer any questions if you do not want to. The interview should take approximately 30 minutes. We will start by discussing the role of WAHO in the pandemic response, followed by coordination and collaboration with other partners, and finally, your recommendations going forward. Given time constraints, we would like to focus on your views of the most important aspects, both successes and areas for improvement.

With your permission I would like to record the interview. The recording will be use by the review team only and will be deleted once the review is complete.

Role of WAHO – c. 20 min.

Thinking about the role WAHO played in supporting your country's COVID-19 response…

Q1. How would you describe WAHO's initial leadership at the beginning of [your country's] pandemic response?

- *Probe, if needed*: visibility, communication, timeliness, coordination; confidence in WAHO guidance, quality of support from WAHO Liaison Officer

Q2. What role has WAHO played in [your country's] COVID-19 response?

- *Probe, if needed*:
  - In your view, how appropriate, timely and relevant was the support?
  - How did WAHO respond to direct requests for support – *what did you ask for and what did you get?*

Q3. Where in particular has WAHO's support added value to your existing capacity or ongoing efforts?

- *Probe, if needed:* Were there aspects of your country's COVID-19 response that would not have been possible or that would have been more challenging without the support from WAHO (e.g. access to PPE, staff deployment, training, etc.)?

Q4. Overall, what could have been improved about WAHO's support in the COVID-19 response?

Communication, Coordination and Collaboration with Partners – c. 5 min.

Q5. Do you think that WAHO helped improve the coordination of international support to your country's COVID-19 response? Please explain.

Q6. How can WAHO improve coordination, communication and collaboration with other partners operating in [your country] and across West Africa?

- *Probe, if needed*: To support in the ongoing COVID-19 response and to support in the medium- and longer-term

Challenges and Recommendations – c. 5 min.

Q7. What recommendations do you have for WAHO, in terms of supporting the ongoing COVID-19 response and recovery, and/or in improving Member States' preparedness and resilience to future pandemics?

- *Probe* on:
  - Local capacity building (support to Member States' health systems strengthening)
  - Further WAHO integration (structural)
  - Strengthening WAHO capacity to better fulfil its mandate (e.g., improving skills, leadership, systems, structures, delivery mechanisms, etc.)
  - Other?
